# Self-reported Clinical Outcomes and Quality of Life in Agammaglobulinemia: the Importance of an Early Diagnosis

**DOI:** 10.1007/s10875-025-01904-z

**Published:** 2025-08-25

**Authors:** Maartje Blom, Annelotte J. Duintjer, Mahnaz Jamee, Melanie de Gier, Markéta Bloomfield, Adam Klocperk, Pavlina Kralickova, Neslihan E. Karaca, Oksana Boyarchuk, Peter Čižnár, Miloš Jeseňák, Svetlana Sharapova, Ekaterina Skopovets, Luis I. Gonzalez-Granado, Serena Palmeri, Stefano Volpi, Andrea Martin Nalda, Sonia Rodriguez Tello, Pere Soler-Palacín, Hassan Abolhassani, Federica Pulvirenti, Bianca Cinicola, Uwe Wintergerst, Godelieve J. de Bree, J. Merlijn van den Berg, Helen L. Leavis, Clementien Vermont, Virgil A.S.H. Dalm, Koen van Aerde, Stefanie Henriet, Hetty Jolink, Judith Potjewijd, Arjan Lankester, Chandoshi Rhea Mukherjee, Dagmar Berghuis, Małgorzata Pac, Benjamin M.J. Shillitoe, Andrew R. Gennery, Mirjam van der Burg

**Affiliations:** 1 Department of Pediatrics, Laboratory for Pediatric Immunology, Willem- Alexander Children’s Hospital, Leiden University Medical Center (LUMC), Albinusdreef 2, Leiden, ZA 2333 the Netherlands; 2 Department of Immunology, Motol University Hospital and 2nd Faculty of Medicine, Charles University, Prague, Czech Republic; 3 Institute of Allergology and Clinical Immunology, Faculty of Medicine, Charles University and University Hospital in Hradec Kralove, Hradec Kralove, Czech Republic; 4 Department of Pediatric Immunology, Ege University Faculty of Medicine, Izmir, Turkey; 5 Department of Children’s Diseases and Pediatric Surgery, I.Horbachevsky Ternopil National Medical University, Ternopil, Ukraine; 6 Paediatric Department, Comenius University Medical Faculty in Bratislava, National Institute for Childhood Diseases, Bratislava, Slovakia; 7 Centre for Inborn Errors of Immunity, Department of Pediatrics, Department of Clinical Immunology and Allergology, Jessenius Faculty of Medicine in Martin, Comenius University in Bratislava, University Hospital in Martin, Martin, Slovakia; 8 Immunology lab, Belarusian Research Center for Pediatric Oncology, Hematology and Immunology, Minsk, Belarus; 9 Primary Immunodeficiencies Unit, Department of Pediatrics, University Hospital 12 octubre, Research Institute imas12 (i+12), Complutense University School of Medicine, Madrid, Spain; 10 Dipartimento di Neuroscienze, Riabilitazione, Oftalmologia, Genetica e Scienze Materno-Infantili (DiNOGMI), Università degli Studi di Genova, Genoa, Italy; 11 Paediatric Rheumatology and Autoinflammatory Diseases Unit, IRCCS Istituto Giannina Gaslini, Genoa, Italy; 12 Pediatric Infectious Diseases and Immunodeficiencies Unit, Children’s Hospital. Vall d’Hebron Barcelona Hospital Campus, Barcelona, Catalonia Spain; 13 Division of Immunology, Department of Medical Biochemistry and Biophysics, Karolinska Institutet, Stockholm, Sweden; 14 Research Center for Immunodeficiencies, Children’s Medical Center, Tehran University of Medical Sciences, Tehran, Iran; 15 Primary Immunodeficiency Unit, Academic hospital Policlinico Umberto I, Rome, Italy; 16 Department of Molecular Medicine, Sapienza University of Rome, Rome, Italy; 17 Department of Maternal Infantile and Urological Sciences, Sapienza University of Rome, Rome, Italy; 18 Department of Pediatrics and Adolescent Medicine, St. Josef Hospital, Braunau, Austria; 19 Department of Infectious Diseases, Amsterdam UMC, Amsterdam, the Netherlands; 20 Department of Paediatric Immunology, Rheumatology and Infectious Diseases, Emma Children’s Hospital, Amsterdam University Medical Centres (AUMC), University of Amsterdam, Amsterdam, the Netherlands; 21 Rheumatology & Clinical Immunology, University Medical Center Utrecht, Utrecht University, Utrecht, the Netherlands; 22 Department of Paediatric Infectious Diseases and Immunology, Erasmus MC Sophia Children’s Hospital, Rotterdam, the Netherlands; 23 Department of Internal Medicine, division of Allergy & Clinical Immunology, Department of Immunology, Erasmus University Medical Center Rotterdam, Rotterdam, the Netherlands; 24 Department of Pediatric Infectious Disease and Immunology, Amalia Children’s Hospital, Radboudumc, Nijmegen, the Netherlands; 25 Department of Infectious Diseases, Leiden University Medical Center, Leiden, the Netherlands; 26 Department of Internal Medicine, division Clinical and Experimental Immunology, Maastricht University Medical Center, Maastricht, the Netherlands; 27 Department of Pediatrics, Division of Pediatric Immunology and Stem Cell Transplantation, Willem-Alexander Children’s Hospital, Leiden, the Netherlands; 28 Worcestershire Acute Hospitals NHS Trust, Worcester, UK; 29 Department of Immunology, Children’s Memorial Health Institute, Warsaw, Poland; 30 Sheffield Children’s NHS Foundation Trust, Sheffield, UK; 31 Translational and Clinical Research Institute, Newcastle University, Newcastle upon Tyne, UK; 32 Great North Children’s Hospital, Newcastle upon Tyne, UK

**Keywords:** X-linked agammaglobulinemia, XLA, B-lymphocyte deficiencies - BTK, KREC, Newborn screening, Quality of life, QoL, Inborn errors of immunity, IEI

## Abstract

**Purpose:**

Patients with (X-linked) agammaglobulinemia (XLA) suffer from severe, recurrent infections potentially leading to life-threatening complications such as sepsis, meningoencephalitis and chronic lung disease. Early diagnosis and timely treatment can prevent infections and secondary complications, emphasizing a role for early detection of XLA via newborn screening (NBS). Our international multicenter survey study aimed to evaluate self-reported outcomes and parental perspectives in XLA patients to determine whether an early diagnosis is associated with better quality of life (QoL).

**Methods:**

QoL-questionnaires included the PedsQL for children and SF-36, CVID_QOL, PADQOL-16 for adults. A new questionnaire was specifically developed for parents about an early diagnosis of XLA.

**Results:**

In total, 88 adult and 65 pediatric XLA patients, and 69 parents from 14 countries completed the survey. Patients with an early diagnosis reported less severe, recurrent infections and less hospitalization (*p* < 0.05). QoL was significantly lower in multiple health domains for pediatric and adult patients with a late diagnosis compared to the general population. Patients with an early diagnosis reported similar QoL outcomes compared to the general population. Parents showed immense support for NBS for XLA stating that an early diagnosis prevents emotional insecurity, health damage, unnecessary diagnostics and allows early access to medical care and informed family planning.

**Conclusion:**

Our study has shown supportive evidence to pursue an early diagnosis of XLA from both a self-reported clinical, health related QoL and parental perspective. The main plea from patients and parents is to achieve an early diagnosis for XLA and severe B-lymphocyte deficiencies with NBS.

**Supplementary Information:**

The online version contains supplementary material available at 10.1007/s10875-025-01904-z.

## Introduction

X-linked agammaglobulinemia (XLA) is an inborn error of immunity (IEI) characterized by defective B-lymphocyte development and severely impaired antibody production [1]. XLA is caused by mutations in the Bruton’s tyrosine kinase (BTK) gene and is the most common cause of congenital agammaglobulinemia, accounting for approximately 85% of cases [2–4]. Autosomal recessive and dominant forms of agammaglobulinemia and other genetic defects affecting early B-lymphocyte development have been reported as well [4, 5]. Patients with severe agammaglobulinemia develop severe, and/or recurrent bacterial infections usually around 4–6 months of age when levels of maternal IgG antibodies decline. Patients are particularly susceptible to infections caused by encapsulated bacteria, most frequently resulting in upper and lower respiratory tract infections, but chronic diarrhea caused by *Giardia lamblia* and susceptibility to enteroviruses and echoviruses, which are implicated in meningo-encephalitis, are also described [1, 6, 7]. Treatment consists of life-long immunoglobulin replacement therapy (IGRT) either intravenously or subcutaneously. In addition, prophylactic antibiotics, careful monitoring and supportive therapies play a role in the treatment of XLA patients [8, 9]. Without treatment, agammaglobulinemia may lead to chronic lung disease (CLD) and irreversible lung damage, which is the main cause of mortality [10–12].

Early detection of IEI in general is important to ensure optimal treatment to avoid infections and non-infectious complications [13, 14]. Recent studies have confirmed that XLA patients with an early diagnosis were less likely to develop lower respiratory tract infections (LRTI), which is of great importance as repeated episodes of these types of infections are highly associated with the development of CLD and bronchiectasis [15–17]. Previous research has additionally shown that after diagnosis and initiation of IGRT, rates of severe invasive infections such as sepsis and meningitis have declined [18]. However, despite appropriate IGRT and supportive therapies, patients are not infection free and continue to experience complications and organ system involvement, suggesting the need for alternative treatment options [16–18]. A recent survey study showed that hematopoietic stem cell transplantation (HSCT) may be an effective and safe alternative treatment option for subgroups of XLA patients [19]. This study, as well as other studies on HSCT in IEI patients, highlight that HSCT before irreversible organ damage has occurred is associated with better clinical outcomes emphasizing an important role for early detection via neonatal screening [19–22].

The chronic context of XLA and severe B-lymphocyte deficiencies pose a potential burden on the life’s of patients, which could be associated with reduced quality of life (QoL). Previous studies have shown lower respiratory-related QoL and a lower general health-related QoL in XLA patients compared to a healthy population, in particular those with bronchiectasis [23]. In addition, having two or more chronic conditions impacted both physical and mental QoL, whereas hospitalization was associated with a significantly decreased physical health QoL [24]. Other studies have shown comparable QoL to the general population, although patients were more absent from school and work compared to the general population [25, 26]. These previous findings raise the question whether an earlier diagnosis would result in fewer chronic conditions or less hospital admissions and higher health-related QoL.

An early diagnosis of XLA and severe B-lymphocyte deficiencies could be realized by the introduction of XLA to newborn screening (NBS) programs. XLA and other severe B-lymphocyte deficiencies can be detected in dried blood spots (DBS) via the quantification of kappa-deleting recombination excision circles (KRECs), an indirect marker for the presence of B-lymphocytes [27, 28]. The primary aim of NBS programs is to identify potentially fatal or disabling conditions in pre-symptomatic newborns for which timely intervention is available and critical to improve the outcome. Nowadays, outcomes should not only be expressed as morbidity and mortality, but disease burden and QoL should also be taken into account. Therefore, our multicenter survey study investigated self-reported outcomes in pediatric and adult XLA patients to determine whether an early diagnosis would lead to improved QoL. In addition, as screening for a disease must be acceptable to participants, healthcare professionals, and the community [29], our study additionally inquires the opinion of parents of XLA patients on early diagnosis of XLA. Parents are key stakeholders in NBS programs and as societal acceptance is a major criterion when introducing new disorders in NBS programs, their support is paramount.

## Methods

### Study Population

Via the European Society for Immunodeficiencies (ESID) Clinical Working Party, European medical centers were invited to join the study from 2021 to 2024. Treating physicians selected their own patients with XLA or severe B-lymphocyte deficiencies (immunophenotype “total absence of B-cells”). XLA patients were also contacted via the local patient organizations with announcements in newsletters, patient magazines, social media and websites. Participation was voluntary and returning the completed questionnaire implied consent. All data was analyzed anonymously. The study was approved by the Medical Ethics Committee of Leiden-Den Haag-Delft (LDD) (reference: N22.018).

### Questionnaire Design and Scoring

Validated questionnaires were used for different age categories including PedsQL for pediatric patients (version 4.0), short-Form-36 (SF-36; version 2.0), CVID_QoL and PADQOL-16 for adults and a new questionnaire regarding early detection of XLA specifically developed for parents [30–33]. The adult and parental questionnaire included generic questions on age of diagnosis, number of infections, hospitalization and treatment. The questionnaire was not intended to collect detailed clinical outcomes and only included self-reported clinical parameters. Questionnaires were translated in English, Dutch, German, French, Polish, Spanish, Italian, Czech, Farsi, Swedish, Turkish, Ukrainian and Portuguese.

A new questionnaire was specifically developed to investigate parental perspectives on early diagnosis of XLA and to evaluate their personal experiences during the diagnostic process. This 27-item questionnaire was developed in collaboration with XLA patients, parents and family members to identify relevant topics and fitting questions. The questionnaire consisted of three sections: (A) General information, (B) Statements on early diagnosis of XLA and (C) Final questions with decisive arguments. A test phase was conducted to check for the concept and wording of questions amongst XLA patients and their families.

### Statistics

Statistical analysis was performed with SPSS version 28.0 for Windows (SPSS, Inc., Chicago, IL, USA). Descriptive statistics were used to describe the characteristics of the participants. Mann-Whitney U or independent t testing was used to compare groups. Cronbach alpha was determined for internal reality and interclass correlation coefficients for agreement between parents proxy and self-reported outcomes. For further details see Supplemental data.

## Results

### Characteristics of the Respondents

**Fig. 1:**
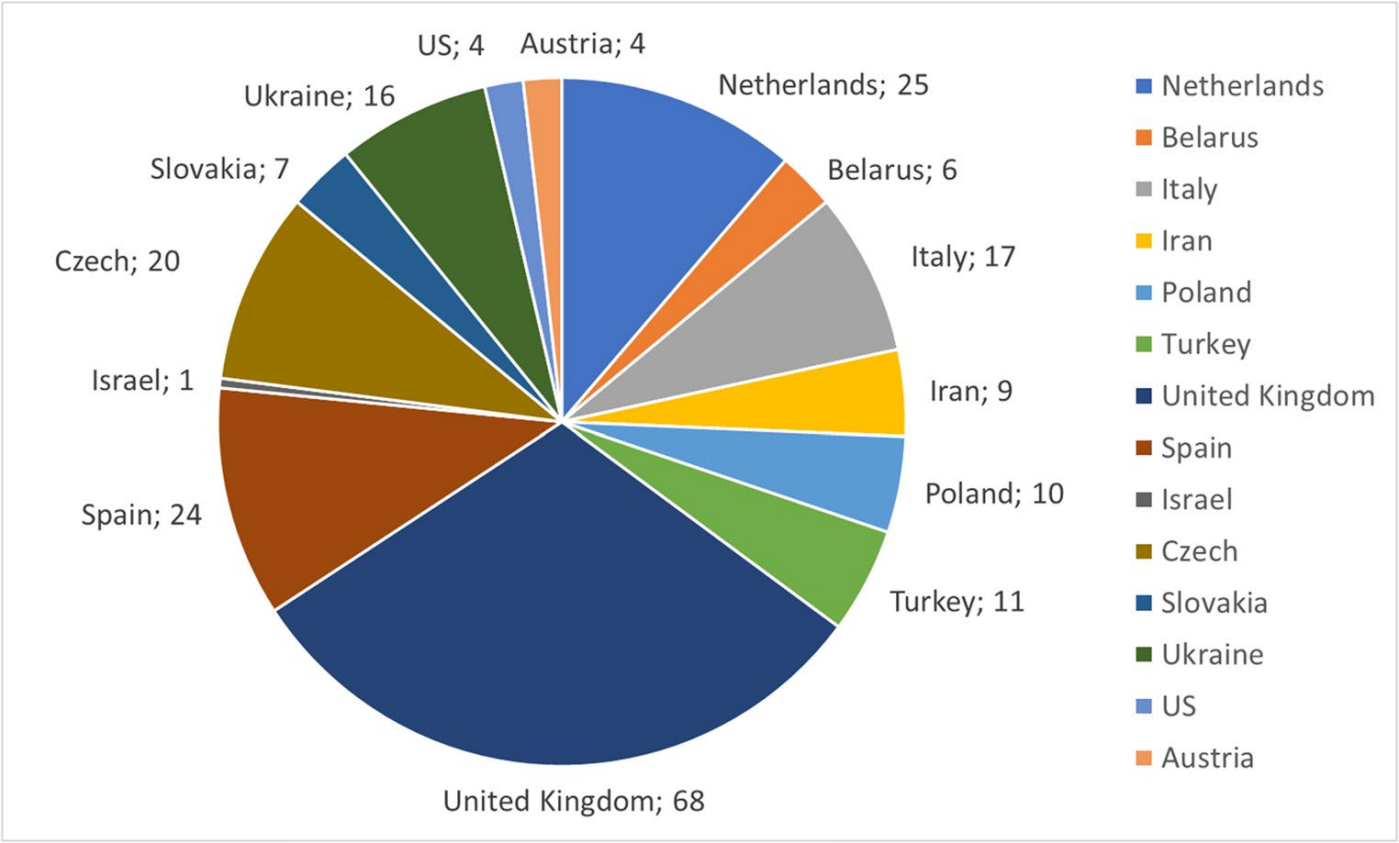
Overview of the participating countries (*N* = 14). The number of completed questionnaires, both adult patients (*N* = 88), pediatric patients (*N* = 65) and parents (*N* = 69) is indicated per country

A total of 153 XLA patients and 69 parents from 14 different countries completed the questionnaires (Fig. 1). Of the XLA patients, 88 were adults with a median age of 31 years (range 18–71 years; Table 1). The pediatric participants were in the age groups of 2–4 years old (*N* = 12), 5–7 years old (*N* = 14), 8–12 years old (*N* = 16), 13–15 years old (*N* = 20) and 16–18 years old (*N* = 3). The median age of onset of symptoms reported by the adult population was 1.0 year (IQR 0.5–28), with a median age at diagnosis of 3.5 years (IQR 1.0–9.0) indicating a diagnostic delay of 2.5 years (Table 1). The median age at the start of therapy was 4.0 years (IQR 1.5–9.0). The median age at onset of symptoms reported by parents was 1.0 year (IQR 0.5–2) and the median age at diagnosis was 2.0 years (IQR 0.9–4) implying a diagnostic delay of 1 year. The average follow-up time equaled the average age when completing the questionnaire (34.2 years; SD 12.8). The parental questionnaire was predominantly completed by mothers (71.2%), one couple completed the questionnaire together and there was one couple who each individually filled out a questionnaire. In 20.3% of the cases, parents had more than one child with XLA.

**Table 1:** General characteristics of the participants. For each category the number of patients/parents (N) and the percentage (%) of the total number are mentioned

**Adult participants**	**Total, ** ***N*** ** = 88**
Age at study, year, mean (SD),	34.2 (12.8)
Age at onset, year, median (IQR)*	1.0 (0.5–2.3)
Age at diagnosis, year, median (IQR)	3.5 (1.0–9.0)
Age at start immunoglobulin replacement therapy, year, median (IQR)*	4.0 (1.5–9.0)
**Pediatric and parent participants**	**Total, ** ***N* = 69**
Parent answering the parental questionnaire	
- Father, *N* (%)	16 (24.2%)
- Mother, *N* (%)	47 (71.2%)
- Father and Mother, *N* (%)	1 (1.5%)
- Legal Guardian, *N* (%)	2 (3.0%)
- Missing	3
Number of children with XLA	
− 1 XLA child, N (%)	51 (79.7%)
− 2 XLA children, N (%)	13 (20.3%)
- Missing	5
Age at onset, year, median (IQR)	1 (0.5–2.0)
Age at diagnosis, year, median (IQR)	2 (0.9–4.0)

*Age at onset and age at the start of the therapy was missing for 43 patients. Age at start of the therapy was not included in the pediatric questionnaire

### Clinical Manifestations

Clinical manifestations reported by adult XLA patients with an early diagnosis (≤ 12 months of age) or a late diagnosis (> 12 months of age) are depicted in Table 2. In total, 79.5% of all patients experienced severe infections (defined as requiring hospital admission, intravenous antibiotics, need of oxygen or fluids to support blood pressure). Recurrent infections (≥2 of the same infections or multiple different types of infection) were reported by 84.6% of patients. The type of infections were mainly upper and lower RTI (29.5% and 70.5% respectively) with bronchiectasis mentioned as a complication by 35.2% of all patients. Other frequently reported types of infections included gastro-intestinal tract infections (6.8%) and eye infections (6.8%). Patients with an early diagnosis reported significantly less infections and less recurrent infections compared to patients with a late diagnosis (*p* < 0.05). Patients with an early diagnosis additionally reported significantly fewer hospital admissions, a known factor associated with QoL, compared to patients with a late diagnosis (*p* = 0.01). Hospitalization was predominantly caused by respiratory tract infections. In total, 30.7% of patients received prophylactic antibiotics; there were no statistical differences in the use of prophylactic antibiotics between patients with an early or late diagnosis.

**Table 2:** Clinical manifestations self-reported by adult XLA patients. For each category the number of adult patients (N) and the percentage (%) of the total number of adult patients are mentioned

	Total, *N* = 88 adult patients*		Early diagnosis (≤ 12 months), *N* = 23 patients	Late diagnosis (> 12 months), *N* = 64 patients	*P*-value
*Severe Infections*,* N(%)*					
- Yes		70 (79.5%)	15 (65.2%)	54 (84.4%)	**0.02**
- No		15 (17.0%)	8 (34.8)	7 (10.9%)	
- I do not know	3 (3.4%)		0	3 (4.7%)	
*Recurrent Infections*, N(%)					
- Yes	66 (84.6%)		10 (52.6%)	55 (94.8%)	**< 0.01**
- No	5 (6.4%)		4 (21.1%)	1 (1.7%)	
- I don’t know	7 (9.0%)		5 (26.3%)	2 (3.4%)	
*Self-reported types of infections or complications*,* N(%)*					
- Lower RTI		62 (70.5%)	11 (47.8%)	50 (78.1%)	**< 0.01**
- Bronchiectasis	31 (35.2%)		7 (30.4%)	23 (35.9%)	NS
- Upper RTI/ENT infections		26 (29.5%)	4 (17.3)	23 (35.9%)	**0.05**
- Gastro-intestinal infection		6 (6.8%)	1 (4.3%)	5 (7.8%)	NS
- Eye infection		6 (6.8%)	1 (4.3%)	5 (7.8%)	NS
- Skin/hair/nail infection		5 (5.6%)	2 (8.6%)	3 (4.7%)	NS
- Unspecified infection		4 (4.6%)	0	4 (6.3%)	NS
- Oral infection		2 (2.3%)	0	2 (3.1%)	NS
- Sepsis		2 (2.3%)	1 (4.3%)	1 (1.6%)	NS
*Hospital Admission*,* N(%)*					
- Yes		32 (66.7%)	4 (33.3%)	28 (77.8%)	**0.01**
- No		14 (29.2%)	7 (58.3%)	7 (19.4%)	
- I don’t know		2 (4.2%)	1 (8.3%)	1 (2.8%)	
*Hospitalization indication*,* N(%)*					
- Lower RTI/Pneumonia	15 (46.9%)		1 (25%)	14 (50%)	NS
- Other respiratory tract infection/ENT infections	5 (15.7)		1 (25%)	4 (14.2%)	NS
- Gastro-intestinal infection		2 (6.3%)	0	2 (7.1)	NS
- Skin/nail infection		2 (6.3%)	1 (25%)	1 (3.6%)	NS
- Osteomyelitis	1 (3.1%)		0	1 (3.6%)	NS
- Not mentioned	10 (31.3%)		1 (25%)	9 (32.1%)	NS
*Prophylactic Antibiotic*,* N(%)*					
- Yes	27 (30.7%)		5 (21.7%)	21 (32.8%)	NS
- No	43 (48.9%)		13 (56.5%)	30 (46.9%)	
- I don’t know	18 (20.5%)		5 (21.7%)	13 (20.3%)	

*One patient had missing data on age at diagnosis. A *p*-value < 0.05 is considered statistically significant. NS = not significant

### Self-Reported QoL by Patients with an Early and Late Diagnosis

Group comparison for QoL outcomes was made between XLA patients with an early diagnosis group defined as ‘diagnosed before the age of 12 months’ and a patients with a late diagnosis diagnosed after 12 months’’. Both groups were compared to the normative population cohorts to investigate differences in self-reported QoL. High internal consistency was found for all questionnaires (Cronbach alpha > 0.70; for more details see Supplemental data).

### Self-Reported QoL in Adult XLA Patients

In total, 88 adult XLA patients completed the SF-36 on their overall health-related QoL. Adult XLA patients reported significantly lower QoL in almost all health domains (with the exception of physical functioning and pain) compared to normative data of a general male population. Patients reported particularly lower general health scores (40.8 versus 73.5, *p* < 0.01) (see Table S1 in the Supplemental data). While patients with an early diagnosis showed similar QoL outcomes when compared to the general population, patients with a late diagnosis reported lower QoL in social functioning and emotional wellbeing (*p* < 0.05; Table 3). A late diagnosis resulted in more psychical and emotional limitations compared to the general population (*p* = 0.01 and *p* = 0.05 respectively). Both patients with an early and late diagnosis expressed lower general health status and vitality or energy. Psychical and mental summary component scores were significantly lower in adult XLA patients with a late diagnosis compared to the general population (*p* < 0.01), whereas for patients with an early diagnosis no differences were observed (Table 3).

**Table 3:** QoL in health domains of the SF-36 reported by adult XLA patients with an early versus late diagnosis. Adult patients are distributed into two groups: patients with an early diagnosis (*N*=23) and late diagnosis (*N*=64). Males from the general normative population (*N*=1055) are included for reference. For each health domain, the SF-36 scores are provided (range 0-100) with standard deviation (SD)

SF-36 health domains	Early Diagnosis (≤ 12 months) *N* = 23 patients*	Late Diagnosis (> 12 months) *N* = 64 patients*	Normative male population (*N* = 1055 males) [34]	*P*-value Early diagnosis vs. late diagnosis	*P*-value Early diagnosis vs. general population	*P*-value Late diagnosis vs. general population
Physical functioning, mean (SD)	89.7 (14.6)	86.7 (19.8)	87.2(21.3)	NS	NS	NS
Physical limitations, mean (SD)	81.9 (21.9)	76.7 (27.4)	86.6 (30.9)	NS	NS	**0.01**
Pain, mean (SD)	82.1 (16.6)	75.4 (22.9)	76.9 (23.0)	NS	NS	NS
General Health, mean (SD)	43.0 (18.5)	39.9 (25.8)	73.5 (20.0)	NS	**< 0.01**	**< 0.01**
Energy Fatigue, mean (SD)	58.3 (22.0)	52.2 (22.9)	63.6 (20.0)	NS	**0.04**	**< 0.01**
Social functioning, mean (SD)	81.0 (26.5)	76.1 (23.7)	85.2 (21.3)	NS	NS	**< 0.01**
Emotional limitations, mean (SD)	77.6 (22.8)	75.5 (27.0)	83.3 (31.3)	NS	NS	**0.05**
Emotional wellbeing, mean (SD)	69.8 (20.5)	63.7 (21.1)	76.4 (17.2)	NS	NS	**< 0.01**
Psychical component summary, mean (SD)	48.8 (8.75)	46.4 (11.2)	50.0 (10.0)	NS	NS	**< 0.01**
Mental component summary, mean (SD)	46.8 (10.9)	44.6 (10.5)	50.0 (10.0)	NS	NS	**< 0.01**

A *p*-value < 0.05 is considered statistically significant. SD, standard deviation; NS, not significant

*Age at diagnosis was missing for one patient

Of all participants, 48 adult XLA patients completed the CVID_Qol questionnaire, which included more IEI specific questions compared to the more generic SF-36. Higher values in the CVID _QoL indicated increased disability in the particular health domains. Adult XLA patients reported significantly more problems with relational functioning compared to a cohort of adult male CVID patients (*p* = 0.05; Table 4) [31]. The health domain relational functioning included topics such as the inability to provide care to loved ones, being afraid to infect others, a tendency to self-isolate, difficulties in sexual relations and difficulty to relate to others. There was a clear trend that adult patients with an early diagnosis scored lower in all health domains of the CVID_QoL indicating less disability, but differences were not statistically significant.

**Table 4:** QoL in health domains of the CVID_QoL reported by adult XLA patients. The number of adult XLA patients (*N*=48) and number of adult male patients with CVID (*N*=46)are indicated. For each health domain, the CVID_QoL scores are provided with standard deviation (SD)

CVID_QoL health domains		Adult XLA patients *N* = 48*	Adult male CVID patients *N* = 46 [31]	*P*-value	Early Diagnosis (≤ 12 months) *N* = 12 XLA patients	Late Diagnosis (> 12 months) *N* = 36 XLA patients	*P*-value
Global score, mean (SD)		28.3 (18.0)	25.7 (14.2)	0.446	25.3 (11.6)	29.4 (19.9)	0.396
Emotional functioning, mean (SD)		30.7 (20.5)	28.5 (15.9)	0.568	26.3 (11.9)	32.3 (22.8)	0.264
Relational functioning, mean (SD)		26.6 (17.3)	20.4 (13.1)	**0.05**	24.5 (14.2)	27.3 (18.4)	0.633
GI and skin system, mean (SD)		20.9 (17.1)	24.2 (19.5)	0.388	17.7 (16.2)	21.9 (17.4)	0.408

*Data was incomplete for 3 patients. A *p*-value < 0.05 is considered statistically significant

Forty-five adult XLA patients completed the PADQOL-16 questionnaire (Table 5). In total, 59% of the patients experienced more fatigue than normal in the past 4 weeks, while 55% expressed having trouble with infections. In contrast, 70% of the patients reported no struggles with keeping up with others and 78% reported not requiring help from others on a frequent basis. Patients had the highest scores in the domain of physical functioning and emotional functioning in agreement with the SF-36 results. The mean total score was 7.95 (SD 6.16) with a range of 0–20 (maximum total score of 32). There are no published data of the PADQOL-16 to compare our cohort to the general population or to other cohorts of PAD patients. Patients with an early diagnosis before the age of 12 months reported significantly fewer problems in the health domain role of physical functioning (*p* = 0.019), which included the item ‘I have missed school or work due to my PID’.

**Table 5:** QoL in health domains of the PADQOL-16 reported by adult XLA patients. The total number of adult XLA patients (*N*=45) are distributed into two groups: patients with an early diagnosis (*N*=12) and late diagnosis (*N*=33). For each health domain, the PADQOL-16 scores (range 0-100) are provided with standard deviation (SD)

PADQOl-16 health domains	Adult XLA patients *N* = 45	Early Diagnosis (≤ 12 months) *N* = 12	Late Diagnosis (> 12 months) *N* = 33	*P*-value
General Health, mean (SD)	70.83 (29.00)	74.24 (23.99)	69.70 (30.75)	0.658
Mental Health, mean (SD)	77.27 (31.35)	86.36 (23.36)	74.24 (33.36)	0.196
Physical Functioning, mean (SD)	70.93 (25.55)	70.45 (28.10)	71.09 (25.10)	0.944
Role of emotional functioning, mean (SD)	76.70 (27.17)	75.00 (31.62)	77.27 (26.04)	0.813
Role of physical functioning, mean (SD)	86.36 (20.50)	95.45 (10.11)	83.33 (22.24)	**0.019**
Social Functioning, mean (SD)	78.98 (24.67)	79.55 (21.85)	79.55 (21.84)	0.758
Vitality, mean (SD)	73.30 (27.17)	68.18 (27.59)	75.00 (27.24)	0.478
Total score, mean (SD).	75.29 (19.45)	76.70 (16.68)	74.80 (20.55)	0.392

A *p*-value < 0.05 is considered statistically significant

### Self-Reported and Rarent-Proxy QoL in Pediatric XLA Patients

Sixty-five pediatric patients aged 2–18 years old completed the PedsQL Generic Core Scales Module. For patients aged 2–4 years (*N* = 14), parents completed the parent-proxy report, while in other age categories patients completed the questionnaire themselves. Additional parent-proxy reports were available for 65% of the patients in other age categories (*N* = 33/51). Both self-reported and parent-proxy QoL of XLA patients were lower in several domains compared to normative data of a general pediatric population [30] (Table S2 in the Supplemental data). Patients with a late diagnosis reported significant lower QoL in all health domains with the exception of social functioning compared to the general pediatric population, while patients with an early diagnosis only reported lower QoL in emotional functioning and school functioning (Table 6). Parents of patients with an early diagnosis reported even higher social functioning compared to the general population (*p* < 0.01). Intraclass correlation coefficients were above 0.70 for all domains with the exception of physical functioning (ICC 0.636), indicating suboptimal agreement on this domain between parents and patients.

**Table 6:** Pediatric self-report and parent-proxy QoL of XLA patients based on PedsQL Generic Core Scales. The total number of pediatric patients XLA patients are distributed into two groups: patients with an early diagnosis (*N*=24) and late diagnosis (*N*=36). For each health domain, the PedsQl scores (range 0-100) are provided with standard deviation (SD)

PedsQL domains	Early Diagnosis (≤ 12 months)*, *N* = 24 patients	Late Diagnosis (> 12 months)*, *N* = 36 patients	General pediatric population *N* = 958 children [30]	*P*-value Early diagnosis vs. late diagnosis	*P*-value Earlydiagnosisvs. general population	*P*-value Late diagnosis vs. general population
Self-total score, mean (SD)	78.9 (11.6)	76.4 (20.5)	82.9 (13.2)	NS	NS	**< 0.01**
Self-total physical health, mean (SD)	85.8 (12.5)	80.4 (20.9)	86.9 (13.9)	NS	NS	**< 0.01**
Self-psychosocial health, mean (SD)	75.2 (12.3)	74.2 (21.7)	80.7 (14.7)	NS	NS	**0.01**
Self-emotional functioning, mean (SD)	70.8 (11.1)	71.6 (23.7)	78.2 (17.4)	NS	**0.04**	**0.03**
Self-social functioning, mean (SD)	89.7 (17.0)	83.0 (23.8)	84.0 (18.6)	NS	NS	NS
Self school functioning, mean (SD)	64.3 (15.7)	68.9 (24.7)	79.9 (16.9)	NS	**< 0.01**	**< 0.01**
Parent total score, mean (SD)	79.5 (9.7)	75.6 (17.3)	81.3 (15.9)	NS	NS	0.04
Parent physical health, mean (SD)	84.7 (13.0)	78.0 (21.6)	83.3 (20.0)	NS	NS	NS
Parent psychosocial health, mean (SD)	76.6 (9.5)	74.2 (19.3)	80.2 (15.8)	NS	**NS**	**0.03**
Parent emotional functioning, mean(SD)	68.4 (11.9)	72.4 (22.2)	80.3 (17.0)	NS	**< 0.01**	**< 0.01**
Parent social functioning, mean (SD)	92.0 (13.7)	82.3 (9.7)	82.2 (20.1)	**0.03**	**0.01**	NS
Parent school functioning, mean (SD)	68.1 (14.7)	67.7 (23.7)	76.9 (20.2)	NS	**0.03**	**< 0.01**

A *p*-value < 0.05 is considered statistically significant. NS, not significant, SD, standard deviation

* Age at diagnosis was missing for five patients

### Parental Perspectives on an Early Diagnose of XLA

Parents were asked to indicate their level of agreement for eighteen statements about the advantages and disadvantages of early detection of XLA and severe B-lymphocyte deficiencies on a 5-point rating scale (Table 7). The statements with the highest level of support indicated that parents would have preferred a diagnosis of their child as soon as possible, even though their child was asymptomatic at that time (rating mean 4.67 and 4.52 respectively). Parents believed that an early diagnosis would have prevented health damage for their child by early access to medical care and the prevention of unnecessary diagnostic tests (rating mean 4.28, 4.54 and 4.29 respectively). Personal quotes from parents stated in Table S3 (Quote 1–4) confirmed these rating means.

**Table 7:** Level of agreement of parents with regard to advantages and disadvantages of early diagnosis of XLA. For each statement, parents indicated their level of agreement on 5-point rating scale (fully disagree to fully agree). The number of parents (N) and percentage of the total number of parents are included as well as the rating mean including the standard deviation (SD)

Statement	Level of agreement^*^ *N* = parents (%)					Rating mean (SD)
	Fully disagree	Disagree	Neutral	Agree	Fully agree	
B1. My child had a long diagnostic process.	7 (12.5)	8 (14.3)	10 (17.9)	14 (25.0)	17 (30.4)	3.46 (1.38)
B2. An early diagnosis could prevent a long period between the first symptoms and the final diagnosis.	2 (2.9)	1 (1.5)	8 (11.8)	12 (17.6)	45 (66.2)	4.43 (0.96)
B3. An early diagnosis could have prevented unnecessary diagnostic tests.	3 (4.5)	2 (3.0)	8 (11.9)	13 (19.4)	41 (61.2)	4.29 (1.08)
B4. In retrospect, I would have preferred to have known the diagnosis shortly after birth, even though my child had no symptoms at that time.	1 (1.5)	3 (4.5)	2 (3.0)	15 (22.4)	46 (68.7)	4.52 (0.87)
B5. The period in which my child had symptoms of XLA/a B-cell deficiency, but had not yet been diagnosed, was a very insecure and (emotionally) stressful period for me.	0 (0)	4 (6.0)	5 (7.5)	16 (23.9)	42 (62.7)	4.43 (0.87)
B6. An early diagnosis would have prevented this uncertain and (emotionally) stressful period.	1 (1.5)	4 (6.2)	6 (9.2)	15 (23.1)	39 (60.0)	4.33 (0.98)
B7. Early medical guidance for me/us as parent(s) would have helped me in this uncertain and (emotionally) stressful period.	1 (1.5)	3 (4.6)	5 (7.7)	16 (24.6)	40 (61.5)	4.40 (0.93)
B8. An early diagnosis would have provided our child with early access to medical counseling and care.	1 (1.5)	2 (2.9)	5 (7.4)	11 (16.2)	49 (72.1)	4.54 (0.87)
B9. An early diagnosis deprives parents of the opportunity to enjoy a seemingly healthy baby in the first months of life.	17 (25.0)	5 (7.4)	10 (14.7)	11 (16.2)	25 (36.8)	3.32 (1.62)
B10. My child would have benefited from early access to medical care and support.	1 (1.5)	0 (0)	9 (13.2)	14 (20.6)	44 (64.7)	4.47 (0.83)
B11. An early diagnosis would have provided me with the opportunity to make informed choices about family planning.	2 (3.0)	3 (4.5)	15 (22.4)	15 (22.4)	32 (47.8)	4.07 (1.07)
B12. If I had known the diagnosis shortly after birth, I would not have been able to enjoy the symptom-free period in my child’s life.	27 (39.7)	14 (20.6)	16 (23.5)	8 (11.8)	3 (4.4)	2.20 (1.21)
B13. An early diagnosis could have prevented serious damage to my child’s health.	1 (1.5)	1 (1.5)	13 (19.4)	15 (22.4)	37 (55.2)	4.28 (0.93)
B14. An early diagnosis would have added little to my child’s quality of life.	23 (34.3)	18 (26.9)	14 (20.9)	5 (7.5)	7 (10.4)	2.32 (1.30)
B15. An early diagnosis would have been a burden in the maternity period with all the information I would receive about my child’s illness.	23 (33.8)	21 (30.9)	10 (14.7)	9 (13.2)	5 (7.4)	2.29 (1.27)
B16. Early detection of B-cell deficiencies makes parents unnecessarily concerned, even before the disease has even manifested itself.	21 (30.9)	22 (32.4)	13 (19.1)	7 (10.3)	5 (7.4)	2.30 (1.22)
B17. XLA and B-cell deficiencies are serious disorders and I wish this disorder could have been detected in my child as soon as possible.	0 (0)	0 (0)	4 (5.9)	14 (20.6)	50 (73.5)	4.67 (0.58)
B18. I think it is important that XLA and other B-cell deficiencies are added to the NBS program.	0 (0)	0 (0)	3 (4.5)	10 (14.9)	54 (80.6)	4.76 (0.52)

*5-point rating scale: 1 fully disagree, 5 fully agree; Missing values are excluded from the percentages

The majority of parents indicated that the period up to diagnosis was very insecure and that an early diagnosis could have prevented this emotional and stressful period (rating mean 4.43 and 4.33 respectively in Table 7; Quotes 4 and 5 in Table S3). The statement indicating that an early diagnosis would deprive parents of the opportunity to enjoy a seemingly health baby in the first months of life (‘golden years’) was met with neutrality (rating mean 3.32). In total, 70% of parents believed that an early diagnosis could provide aid in family planning (Quote 7 and 8 in Table S3).

Parents did not agree that an early diagnosis would have added little to their child’s QoL (rating mean 2.32) stating that an early diagnosis would limit health damage, mental burden and improve QoL (Quote 9 and 10 in Table S3). Parents did not agree that an early diagnosis would result in unnecessary concerns before manifestation of the disease (rating mean 2.30) or that an early diagnosis would be a burden in the maternity period (rating mean 2.29; Quote 11, Table S3). The vast majority of parents expressed high support for the inclusion of XLA and other B-lymphocyte deficiencies in the NBS program (rating mean 4.76; Quote 12).

In free text boxes, parents mentioned other important arguments for an early diagnosis of XLA that were not included in the statements such as raising awareness amongst clinicians (Table S3, Quote 13 and 14) and preventing potential morbidity of undiagnosed XLA patients (Table S3, Quote 15).

## Discussion

XLA and other severe B-lymphocyte deficiencies are chronic disorders with severe impact on the life’s of patients and parents. Earlier diagnosis and timely initiation of therapy could potentially reduce morbidity and mortality in these patients. The purpose of this study was to gain insight into the QoL of pediatric and adult XLA patients and to see if an earlier diagnosis would result in improved self-reported outcomes. Our international survey study is the largest study to date on QoL in XLA patients, taking both the patient and parental perspectives into account.

Patients with an early diagnosis (≤ 12 months of age) reported significantly fewer severe infections and recurrent infections in their lifetime compared to patients with a late diagnosis (*p* < 0.05). Recurrent LRTIs are highly associated with the development of CLD, the leading cause of morbidity and mortality in XLA patients [11, 15, 16]. Early diagnosis could therefore lead to significant health benefits in these patients. In addition, patients with an early diagnosis reported less hospital admissions, a known factor associated with lower QoL [24, 35]. As these data were self-reported and therefore subjective measurements, there is need for a confirmatory study that includes more detailed clinical parameters collected by health care professionals. These study results will be able to objectively determine the exact health gain of patients with an early diagnosis compared to a late diagnosis in terms of morbidity and mortality.

Physicians face several challenges when diagnosing XLA patients, potentially in resulting in a diagnostic delay [12, 36]. The mean diagnostic delay in our study population was 1.0 year in pediatric patients (reported by parents) and 2.5 years the adult patient population (self-reported). Other studies have reported diagnostic delays ranging from less than 12 months to over 3 years [10, 12, 37]. While the age at diagnosis has reduced dramatically over the last 20 years, this improvement is now reaching a plateau at 2 to 3.5 years [16]. A reduction in diagnostic delay was observed after the introduction of a nationwide registry potentially by creating more awareness among physicians [11]. Raising awareness and improving knowledge about clinical manifestations of XLA was a topic frequently mentioned by the adult patients and parents in our study. Securing a diagnosis before the age of 12 months on clinical grounds is extremely challenging. NBS might be the only feasible option to bring the age at diagnosis further down. However, educational programs and public awareness campaigns might be a more feasible option for some countries in the direct future to enable an early diagnosis and timely initiation of IGRT [38]. Close partnerships of health professionals and sharing experiences internationally could help to improve outcomes for XLA patients on a global level.

Our study showed that pediatric XLA patients have significantly lower QoL scores compared to the general population, especially patients with a late diagnosis. Previous cohorts with pediatric patients have confirmed similar results in PAD patients [39–41]. Both children and parents in our study reported more problems in the health domains of school functioning, emotional functioning and psychosocial health. In the study of Klungland-Berg et al.., 40% of children with antibody deficiency lost more than 10 days a year of school, while 20% lost over 25 days due to IGRT [41]. Soresine et al.. suggested that parents might consider their child with XLA as more fragile, needing more protection and thereby influencing autonomy, resulting in more problems with emotional and school functioning [40]. In the recent study by Hernandez-Truijllo et al., neurological complications such as cognitive disabilities, speech delay and difficulties with educational academic activities were reported in more than 35% of their cohort of XLA patients [18]. Neurologic manifestations of XLA are an important co-morbidity and, although not yet fully understood, could be linked to the reduced QoL outcomes in psychosocial health domains.

Although some studies showed no differences in QoL between adult XLA patients and the general population [25, 26], our study showed significantly lower QoL in adult XLA patients in almost all health domains with the exception of pain and physical functioning. It should be noted that the SF-36 is a more generic QoL instrument, which might over- or underestimate the true impact of a chronic disease. The results of the PADQOl-16 showed lower QoL scores in the health domain ‘role of physical functioning’ in patients with a late diagnosis. The role of psychical functioning included missing school or work due to their disease, which was also mentioned by the study of Winkelstein et al.. where 65% of the XLA patients missed work or school due to illness during the past year, compared to 46% of the general population of adults [25].

Although the number of included patients was relatively high for a study concerning a rare disease, the smaller groups per age category and early versus late diagnosis have potentially limited the power for comparisons between groups. By reaching out to multiple clinical centers across the world, optimization of sample size was pursued and the study population was more representative for XLA patients around the globe. Potential bias was however introduced, as surveys are usually skewed towards healthy participants potentially resulting in higher QoL scores. In addition, as diagnostic strategies and treatment option might vary between countries, for future studies, it would be advised to compare the results from the XLA group to matched control groups instead of normative values for the standardized measures.

Patients and parents favored an earlier diagnosis to facilitate earlier initiation of treatment. However, as patients with an early diagnosis did not show better QoL in all health domains compared to patients with a late diagnosis, clinical management might not be satisfactory in XLA patients. Lifelong IGRT is not sufficient to control morbidity and mortality in XLA patients and IGRT itself might be associated with lower QoL [11, 42, 43]. Other treatment modalities such as HSCT or gene therapy should be considered as alternative treatment options for selected XLA patients. HSCT is not routinely used in XLA patients as it is unclear whether benefits outweigh the risks of this invasive treatment in XLA patients. A recent study showed that HSCT might be a salvage treatment in XLA patients with complications such as refractory infections or hematological malignancies, although HSCT before to irreversible organ damage would be the preferred option [9, 19]. This might be supported by the fact that T-cell abnormalities and abnormal cell-mediated immune responses have also been described in XLA patients [44]. Whether HSCT should be routinely offered to XLA patients remains a topic of debate. For many IEIs, gene therapy seems to be a promising curative treatment modality in the near future [45, 46]. With reduced-intensity/toxicity conditioning regimes and no risk of graft-versus-host-disease (GVHD), gene therapy might be an eloquent treatment option for XLA patients. Proof of concept studies in murine models of XLA have been proven to be effective and research groups are now working toward clinical trials in human patients [47–49].The personal stories of parents of XLA patients have emphasized the support for an early diagnosis of XLA and severe B-lymphocyte deficiencies via NBS. Our findings go hand in hand with previous studies that have shown a positive attitude of parents towards the expansion of NBS programs with new disorders [50–52]. The perspective of parents as key stakeholders in NBS is of great value for policymakers who aim to balance the advantages and disadvantages of early detection of rare hereditary disorders.

In conclusion, our study has shown supportive evidence to pursue an early diagnosis of XLA from both a self-reported clinical, health-related QoL and parental perspective. Adult XLA patients with an early diagnosis reported less severe and/or recurrent infections and less hospitalization. Both pediatric and adult patients have significantly lower QoL as compared to the general population, with lower QoL scores in some health domains for patients with a late diagnosis versus early diagnosis. A next study should confirm these self-reported data with more detailed clinical data collected from patients records. More awareness amongst physicians in recognizing early manifestations of XLA remains of utmost importance, but the main plea from patients and parents is to achieve an early diagnosis via NBS for XLA and severe B-lymphocyte deficiencies.

## Supplementary Information

Below is the link to the electronic supplementary material.

ESM 1 DOCX (24.6 KB)

## Data Availability

Data is provided within the manuscript or supplementary information files. The raw datasets generated during and/or analysed during the current study are available from the corresponding author on reasonable request.

## References

[1] Lackey AE, Ahmad F. X-Linked Agammaglobulinemia. 2023 Jul 3. In: StatPearls [Internet]. Treasure Island (FL): StatPearls Publishing; 2025. 31751055

[2] Tsukada S, Saffran DC, Rawlings DJ, Parolini O, Allen RC, Klisak I, et al. Deficient expression of a B cell cytoplasmic tyrosine kinase in human X-linked agammaglobulinemia. Cell. 1993;72(2):279–90. 8425221 10.1016/0092-8674(93)90667-f

[3] Vetrie D, Vorechovský I, Sideras P, Holland J, Davies A, Flinter F, et al. The gene involved in X-linked agammaglobulinaemia is a member of the Src family of protein-tyrosine kinases. Nature. 1993;361(6409):226–33. 8380905 10.1038/361226a0

[4] Cardenas-Morales M, Hernandez-Trujillo VP. Agammaglobulinemia: from X-linked to autosomal forms of disease. Clin Rev Allergy Immunol. 2022;63(1):22–35. 34241796 10.1007/s12016-021-08870-5 PMC8269404

[5] Bousfiha A, Moundir A, Tangye SG, Picard C, Jeddane L, Al-Herz W, et al. The 2022 update of IUIS phenotypical classification for human inborn errors of immunity. J Clin Immunol. 2022;42(7):1508–20. 36198931 10.1007/s10875-022-01352-z

[6] Conley ME, Rohrer J, Minegishi Y. X-linked agammaglobulinemia. Clin Rev Allergy Immunol. 2000;19(2):183–204. 11107501 10.1385/CRIAI:19:2:183

[7] Morosky S, Wells AI, Lemon K, Evans AS, Schamus S, Bakkenist CJ, et al. The neonatal Fc receptor is a pan-echovirus receptor. Proc Natl Acad Sci U S A. 2019;116(9):3758–63. 30808762 10.1073/pnas.1817341116 PMC6397586

[8] Moschese V, Martire B, Soresina A, Chini L, Graziani S, Monteferrario E, et al. Anti-infective prophylaxis for primary immunodeficiencies: what is done in Italian primary immunodeficiency network centers (IPINet) and review of the literature. J Biol Regul Homeost Agents. 2013;27(4):935–46. 24382174

[9] Shillitoe BMJ, Gennery AR. An update on X-Linked agammaglobulinaemia: clinical manifestations and management. Curr Opin Allergy Clin Immunol. 2019;19(6):571–7. 31464718 10.1097/ACI.0000000000000584

[10] Abolhassani H, Hirbod-Mobarakeh A, Shahinpour S, Panahi M, Mohammadinejad P, Mirminachi B, et al. Mortality and morbidity in patients with X-linked agammaglobulinaemia. Allergol Immunopathol (Madr). 2015;43(1):62–6. 24485939 10.1016/j.aller.2013.09.013

[11] Lougaris V, Soresina A, Baronio M, Montin D, Martino S, Signa S, et al. Long-term follow-up of 168 patients with X-linked agammaglobulinemia reveals increased morbidity and mortality. J Allergy Clin Immunol. 2020;146(2):429–37. 32169379 10.1016/j.jaci.2020.03.001

[12] El-Sayed ZA, Abramova I, Aldave JC, Al-Herz W, Bezrodnik L, Boukari R, et al. X-linked agammaglobulinemia (XLA):Phenotype, diagnosis, and therapeutic challenges around the world. World Allergy Organ J. 2019;12(3):100018. 30937141 10.1016/j.waojou.2019.100018 PMC6439403

[13] Routes J, Abinun M, Al-Herz W, Bustamante J, Condino-Neto A, De La Morena MT, et al. ICON: the early diagnosis of congenital immunodeficiencies. J Clin Immunol. 2014;34(4):398–424. 24619621 10.1007/s10875-014-0003-x

[14] Chun JK, Lee TJ, Song JW, Linton JA, Kim DS. Analysis of clinical presentations of Bruton disease: a review of 20 years of accumulated data from pediatric patients at severance hospital. Yonsei Med J. 2008;49(1):28–36. 18306466 10.3349/ymj.2008.49.1.28 PMC2615253

[15] O’Toole D, Groth D, Wright H, Bonilla FA, Fuleihan RL, Cunningham-Rundles C, et al. X-Linked agammaglobulinemia: infection frequency and infection-Related mortality in the USIDNET registry. J Clin Immunol. 2022;42(4):827–36. 35288819 10.1007/s10875-022-01237-1 PMC8920804

[16] Plebani A, Soresina A, Rondelli R, Amato GM, Azzari C, Cardinale F, et al. Clinical, immunological, and molecular analysis in a large cohort of patients with X-linked agammaglobulinemia: an Italian multicenter study. Clin Immunol. 2002;104(3):221–30. 12217331 10.1006/clim.2002.5241

[17] Winkelstein JA, Marino MC, Lederman HM, Jones SM, Sullivan K, Burks AW, et al. X-Linked agammaglobulinemia: report on a united States registry of 201 patients. Medicine. 2006;85(4):193–202. 16862044 10.1097/01.md.0000229482.27398.ad

[18] Hernandez-Trujillo V, Zhou C, Scalchunes C, Ochs HD, Sullivan KE, Cunningham-Rundles C, et al. A registry study of 240 patients with X-Linked agammaglobulinemia living in the USA. J Clin Immunol. 2023;43(6):1468–77. 37219739 10.1007/s10875-023-01502-x PMC10354121

[19] Nishimura A, Uppuluri R, Raj R, Swaminathan VV, Cheng Y, Abu-Arja RF, et al. An international survey of allogeneic hematopoietic cell transplantation for X-Linked agammaglobulinemia. J Clin Immunol. 2023;43(8):1827–39. 37454339 10.1007/s10875-023-01551-2

[20] Albert MH, Slatter MA, Gennery AR, Güngör T, Bakunina K, Markovitch B, et al. Hematopoietic stem cell transplantation for Wiskott-Aldrich syndrome: an EBMT inborn errors working party analysis. Blood. 2022;139(13):2066–79. 35100336 10.1182/blood.2021014687

[21] Lankester AC, Neven B, Mahlaoui N, von Asmuth EGJ, Courteille V, Alligon M, et al. Hematopoietic cell transplantation in severe combined immunodeficiency: the SCETIDE 2006–2014 European cohort. J Allergy Clin Immunol. 2022;149(5):1744–e548. 34718043 10.1016/j.jaci.2021.10.017

[22] Chiesa R, Wang J, Blok HJ, Hazelaar S, Neven B, Moshous D, et al. Hematopoietic cell transplantation in chronic granulomatous disease: a study of 712 children and adults. Blood. 2020;136(10):1201–11. 32614953 10.1182/blood.2020005590

[23] Bryan BA, Battersby A, Shillitoe BM, Barge D, Bourne H, Flood T, et al. Respiratory health and related quality of life in patients with congenital agammaglobulinemia in the Northern region of the UK. J Clin Immunol. 2016;36(5):472–9. 27091141 10.1007/s10875-016-0284-3 PMC4896978

[24] Altman K, Zhou C, Hernandez-Trujillo V, Scalchunes C, Rawlings DJ, de la Morena MT. Health-Related quality of life in 91 patients with X-Linked agammaglobulinemia. J Clin Immunol. 2022;42(4):811–8. 35284987 10.1007/s10875-022-01222-8

[25] Winkelstein JA, Conley ME, James C, Howard V, Boyle J. Adults with X-linked agammaglobulinemia: impact of disease on daily lives, quality of life, educational and socioeconomic status, knowledge of inheritance, and reproductive attitudes. Med (Baltim). 2008;87(5):253–8. 10.1097/MD.0b013e318187ed81 PMC2831411 18794707

[26] Howard V, Greene JM, Pahwa S, Winkelstein JA, Boyle JM, Kocak M, et al. The health status and quality of life of adults with X-linked agammaglobulinemia. Clin Immunol. 2006;118(2–3):201–8. 16377251 10.1016/j.clim.2005.11.002

[27] Borte S, von Döbeln U, Fasth A, Wang N, Janzi M, Winiarski J, et al. Neonatal screening for severe primary immunodeficiency diseases using high-throughput triplex real-time PCR. Blood. 2012;119(11):2552–5. 22130802 10.1182/blood-2011-08-371021

[28] van Zelm MC, van der Burg M, Langerak AW, van Dongen JJ. PID comes full circle: applications of V(D)J recombination excision circles in research, diagnostics and newborn screening of primary immunodeficiency disorders. Front Immunol. 2011;2:12. 22566803 10.3389/fimmu.2011.00012 PMC3342366

[29] Kelleher KJ, Gardner W, Kemper AR, Chavez L, Pajer K, Rosic T. Principles for primary care screening in the context of population health. Acad Pediatr. 2024. 10.1016/j.acap.2024.02.015. 10.1016/j.acap.2024.02.015 38458490

[30] Varni JW, Seid M, Kurtin PS. PedsQL 4.0: reliability and validity of the pediatric quality of life inventory version 4.0 generic core scales in healthy and patient populations. Med Care. 2001;39(8):800–12. 11468499 10.1097/00005650-200108000-00006

[31] Quinti I, Pulvirenti F, Giannantoni P, Hajjar J, Canter DL, Milito C, et al. Development and initial validation of a questionnaire to measure Health-Related quality of life of adults with common variable immune deficiency: the cvid_qol questionnaire. J Allergy Clin Immunol Pract. 2016;4(6):1169–e794. 27665385 10.1016/j.jaip.2016.07.012

[32] Andersen JB, Midttun K, Feragen KJB. Measuring quality of life of primary antibody deficiency patients using a disease-specific health-related quality of life questionnaire for common variable immunodeficiency (CVID_QoL). J Patient Rep Outcomes. 2019;3(1):15. 30806830 10.1186/s41687-019-0101-x PMC6391500

[33] Ballow M, Conaway MR, Sriaroon P, Rachid RA, Seeborg FO, Duff CM et al. Construction and validation of a novel disease-specific quality-of-life instrument for patients with primary antibody deficiency disease (PADQOL-16). J Allergy Clin Immunol. 2017;139(6):2007-10.e8. 10.1016/j.jaci.2016.11.029 28065678

[34] Ware JE Jr., Sherbourne CD. The MOS 36-item short-form health survey (SF-36). I. Conceptual framework and item selection. Med Care. 1992;30(6):473–83. 1593914

[35] Carrillo-Tapia E, García-García E, Herrera-González NE, Yamazaki-Nakashimada MA, Staines-Boone AT, Segura-Mendez NH, et al. Delayed diagnosis in X-linked agammaglobulinemia and its relationship to the occurrence of mutations in BTK non-kinase domains. Expert Rev Clin Immunol. 2018;14(1):83–93. 29202590 10.1080/1744666X.2018.1413349

[36] Chen XF, Wang WF, Zhang YD, Zhao W, Wu J, Chen TX. Clinical characteristics and genetic profiles of 174 patients with X-linked agammaglobulinemia: report from shanghai, China (2000–2015). Med (Baltim). 2016;95(32):e4544. 10.1097/MD.0000000000004544 PMC4985333 27512878

[37] El-Sayed ZA, Radwan N. Newborn screening for primary immunodeficiencies: the gaps, challenges, and outlook for developing countries. Front Immunol. 2019;10:2987. 32082296 10.3389/fimmu.2019.02987 PMC7002357

[38] Titman P, Allwood Z, Gilmour C, Malcolmson C, Duran-Persson C, Cale C, et al. Quality of life in children with primary antibody deficiency. J Clin Immunol. 2014;34(7):844–52. 25005831 10.1007/s10875-014-0072-x PMC4165866

[39] Soresina A, Nacinovich R, Bomba M, Cassani M, Molinaro A, Sciotto A, et al. The quality of life of children and adolescents with X-linked agammaglobulinemia. J Clin Immunol. 2009;29(4):501–7. 19089603 10.1007/s10875-008-9270-8

[40] Berg AK, Diseth TH, Abrahamsen TG, Halvorsen K, Reinfjell T, Erichsen HC. Primary antibody deficiency: the impact on the quality of life and mental health of affected children and their parents. Acta Paediatr. 2021;110(5):1645–52. 33420742 10.1111/apa.15752

[41] Anderson JT, Cowan J, Condino-Neto A, Levy D, Prusty S. Health-related quality of life in primary immunodeficiencies: impact of delayed diagnosis and treatment burden. Clin Immunol. 2022;236:108931. 35063670 10.1016/j.clim.2022.108931

[42] Jones GL, Vogt KS, Chambers D, Clowes M, Shrimpton A. What is the burden of Immunoglobulin replacement therapy in adult patients with primary immunodeficiencies?? A systematic review. Front Immunol. 2018;9. 10.3389/fimmu.2018.01308. 10.3389/fimmu.2018.01308 PMC6043812 30034388

[43] Chawla S, Jindal AK, Arora K, Tyagi R, Dhaliwal M, Rawat A. T cell abnormalities in X-Linked agammaglobulinaemia: an updated review. Clin Rev Allergy Immunol. 2023;65(1):31–42. 35708830 10.1007/s12016-022-08949-7 PMC9201264

[44] Moreau T, Calmels B, Barlogis V, Michel G, Tonnelle C, Chabannon C. Potential application of gene therapy to X-linked agammaglobulinemia. Curr Gene Ther. 2007;7(4):284–94. 17969561 10.2174/156652307781369128

[45] Ott de Bruin LM, Lankester AC, Staal FJT. Advances in gene therapy for inborn errors of immunity. Curr Opin Allergy Clin Immunol. 2023;23(6):467–77. 37846903 10.1097/ACI.0000000000000952 PMC10621649

[46] Bestas B, Turunen JJ, Blomberg KEM, Wang Q, Månsson R, El Andaloussi S, et al. Splice-Correction strategies for treatment of X-Linked agammaglobulinemia. Curr Allergy Asthma Rep. 2015;15(3):4. 25638286 10.1007/s11882-014-0510-0 PMC4312560

[47] Gray DH, Villegas I, Long J, Santos J, Keir A, Abele A, et al. Optimizing integration and expression of Transgenic bruton’s tyrosine kinase for CRISPR-Cas9-Mediated gene editing of X-Linked agammaglobulinemia. Crispr J. 2021;4(2):191–206. 33876953 10.1089/crispr.2020.0080 PMC8336228

[48] Seymour BJ, Singh S, Certo HM, Sommer K, Sather BD, Khim S, et al. Effective, safe, and sustained correction of murine XLA using a UCOE-BTK promoter-based lentiviral vector. Mol Ther Methods Clin Dev. 2021;20:635–51. 33718514 10.1016/j.omtm.2021.01.007 PMC7907679

[49] Blom M, Bredius RGM, Jansen ME, Weijman G, Kemper EA, Vermont CL, et al. Parents’ perspectives and societal acceptance of implementation of newborn screening for SCID in the Netherlands. J Clin Immunol. 2021;41(1):99–108. 33070266 10.1007/s10875-020-00886-4 PMC7846522

[50] Joseph G, Chen F, Harris-Wai J, Puck JM, Young C, Koenig BA. Parental views on expanded newborn screening using Whole-Genome sequencing. Pediatrics. 2016;137(Suppl 1Suppl 1):S36–46. 26729702 10.1542/peds.2015-3731H PMC4939888

[51] DeLuca JM. Public attitudes toward expanded newborn screening. J Pediatr Nurs. 2018;38:e19–23. 29033105 10.1016/j.pedn.2017.10.002

[52] Albuquerque de Almeida F, Al MJ, Koymans R, Riistama J, Pauws S, Severens JL. Impact of hospitalisation on health-related quality of life in patients with chronic heart failure. Health Qual Life Outcomes. 2020;18(1):262. 32746842 10.1186/s12955-020-01508-8 PMC7397623

