# Correction to: Self-reported Clinical Outcomes and Quality of Life in Agammaglobulinemia: the Importance of an Early Diagnosis

**DOI:** 10.1007/s10875-025-01944-5

**Published:** 2025-10-09

**Authors:** Maartje Blom, Annelotte J. Duintjer, Mahnaz Jamee, Melanie de Gier, Markéta Bloomfield, Adam Klocperk, Pavlina Kralickova, Neslihan E. Karaca, Oksana Boyarchuk, Peter Čižnár, Miloš Jeseňák, Svetlana Sharapova, Ekaterina Skopovets, Luis I. Gonzalez-Granado, Serena Palmeri, Stefano Volpi, Andrea Martin Nalda, Sonia Rodriguez Tello, Pere Soler-Palacín, Hassan Abolhassani, Federica Pulvirenti, Bianca Cinicola, Uwe Wintergerst, Godelieve J. de Bree, J. Merlijn van den Berg, Helen L. Leavis, Clementien Vermont, Virgil A.S.H. Dalm, Koen van Aerde, Stefanie Henriet, Hetty Jolink, Judith Potjewijd, Arjan Lankester, Chandoshi Rhea Mukherjee, Dagmar Berghuis, Małgorzata Pac, Benjamin M.J. Shillitoe, Andrew R. Gennery, Mirjam van der Burg

**Affiliations:** 1 Department of Pediatrics, Laboratory for Pediatric Immunology, Willem- Alexander Children’s Hospital, Leiden University Medical Center (LUMC), Albinusdreef 2, Leiden, ZA 2333 the Netherlands; 2 Department of Immunology, Motol University Hospital and 2nd Faculty of Medicine, Charles University, Prague, Czech Republic; 3 Institute of Allergology and Clinical Immunology, Faculty of Medicine, Charles University and University Hospital in Hradec Kralove, Hradec Kralove, Czech Republic; 4 Department of Pediatric Immunology, Ege University Faculty of Medicine, Izmir, Turkey; 5 Department of Children’s Diseases and Pediatric Surgery, I.Horbachevsky Ternopil National Medical University, Ternopil, Ukraine; 6 Paediatric Department, Comenius University Medical Faculty in Bratislava, National Institute for Childhood Diseases, Bratislava, Slovakia; 7 Centre for Inborn Errors of Immunity, Department of Pediatrics, Department of Clinical Immunology and Allergology, Jessenius Faculty of Medicine in Martin, Comenius University in Bratislava, University Hospital in Martin, Martin, Slovakia; 8 Immunology lab, Belarusian Research Center for Pediatric Oncology, Hematology and Immunology, Minsk, Belarus; 9 Primary Immunodeficiencies Unit, Department of Pediatrics, University Hospital 12 octubre, Research Institute imas12 (i+12), Complutense University School of Medicine, Madrid, Spain; 10 Dipartimento di Neuroscienze, Riabilitazione, Oftalmologia, Genetica e Scienze Materno-Infantili (DiNOGMI), Università degli Studi di Genova, Genoa, Italy; 11 Paediatric Rheumatology and Autoinflammatory Diseases Unit, IRCCS Istituto Giannina Gaslini, Genoa, Italy; 12 Pediatric Infectious Diseases and Immunodeficiencies Unit, Children’s Hospital. Vall d’Hebron Barcelona Hospital Campus, Barcelona, Catalonia, Spain; 13 Division of Immunology, Department of Medical Biochemistry and Biophysics, Karolinska Institutet, Stockholm, Sweden; 14 Research Center for Immunodeficiencies, Children’s Medical Center, Tehran University of Medical Sciences, Tehran, Iran; 15 Primary Immunodeficiency Unit, Academic hospital Policlinico Umberto I, Rome, Italy; 16 Department of Molecular Medicine, Sapienza University of Rome, Rome, Italy; 17 Department of Maternal Infantile and Urological Sciences, Sapienza University of Rome, Rome, Italy; 18 Department of Pediatrics and Adolescent Medicine, St. Josef Hospital, Braunau, Austria; 19 Department of Infectious Diseases, Amsterdam UMC, Amsterdam, the Netherlands; 20 Department of Paediatric Immunology, Rheumatology and Infectious Diseases, Emma Children’s Hospital, Amsterdam University Medical Centres (AUMC), University of Amsterdam, Amsterdam, the Netherlands; 21 Rheumatology & Clinical Immunology, University Medical Center Utrecht, Utrecht University, Utrecht, the Netherlands; 22 Department of Paediatric Infectious Diseases and Immunology, Erasmus MC Sophia Children’s Hospital, Rotterdam, the Netherlands; 23 Department of Internal Medicine, division of Allergy & Clinical Immunology, Department of Immunology, Erasmus University Medical Center Rotterdam, Rotterdam, the Netherlands; 24 Department of Pediatric Infectious Disease and Immunology, Amalia Children’s Hospital, Radboudumc, Nijmegen, the Netherlands; 25 Department of Infectious Diseases, Leiden University Medical Center, Leiden, the Netherlands; 26 Department of Internal Medicine, division Clinical and Experimental Immunology, Maastricht University Medical Center, Maastricht, the Netherlands; 27 Department of Pediatrics, Division of Pediatric Immunology and Stem Cell Transplantation, Willem-Alexander Children’s Hospital, Leiden, the Netherlands; 28 Worcestershire Acute Hospitals NHS Trust, Worcester, UK; 29 Department of Immunology, Children’s Memorial Health Institute, Warsaw, Poland; 30 Sheffield Children’s NHS Foundation Trust, Sheffield, UK; 31 Translational and Clinical Research Institute, Newcastle University, Newcastle upon Tyne, UK; 32 Great North Children’s Hospital, Newcastle upon Tyne, UK

**Correction to: Journal of Clinical Immunology**

10.1007/s10875-025-01904-z

The original version of this article had Pere Soler-Palacín’s name formatted incorrectly. It should be “Soler-Palacín P.” instead of “Palacin P.S.“.

Correct Formatting:

From:

< GivenName > Pere</GivenName>

< GivenName > Soler</GivenName>

< FamilyName > Palacín</FamilyName>

To:

< GivenName > Pere </GivenName>

< FamilyName > Soler-Palacín</FamilyName>

The original version has been corrected.

