# Supplementary material for: Self-reported Clinical Outcomes and Quality of Life in Agammaglobulinemia: the Importance of an Early Diagnosis
Source: J Clin Immunol. 2025 Aug 25;45(1):125. doi: 10.1007/s10875-025-01904-z (PMC12378137; doi:10.1007/s10875-025-01904-z)
Supplement: Supplementary file 1 — DOCX (24.6 KB) [file 10875_2025_1904_MOESM1_ESM.docx]

**Supplemental data**

**Methods**

**Questionnaire design and scoring**

Validated questionnaires were used for different age categories including PedsQL for pediatric patients (version 4.0), Short-Form-36 (SF-36; version 2.0), CVID_QoL and PADQOL-16 for adults and a new questionnaire regarding early detection of XLA specifically developed for parents. The adult and parental questionnaire included generic questions on age of diagnosis, number of infections, hospitalization and treatment. The questionnaire was not intended to collect detailed clinical outcomes and only included self-reported clinical parameters. Questionnaires were translated in English, Dutch, German, French, Polish, Spanish, Italian, Czech, Farsi, Swedish, Turkish, Ukrainian and Portuguese.

Standardized questionnaires were used to investigate health related QoL in XLA patients. The PedsQL Generic Core Scales Module was used for pediatric patients age 2-18 years including child self-reports and parent-proxy reports. For the age range (age 2-4) only a parent proxy-report was available. A 5-point response scale is used (0 = never a problem, 4 = almost always a problem). Items are reverse-scored and linearly transformed to a 0 to 100 scale, so that higher scores indicate better health-related QoL. The PedsQL consists of 23 items on four domains: Physical, Emotional, Social and School Functioning leading to Scale Scores, a Psychosocial Health Summary Score and a Total Score [29].

For adult XLA patients (>18 years and older), the SF-36 questionnaire was used which comprises of 36 items categorized into eight domains: physical functioning (10 items); physical role limitations (four items); bodily pain (two items); general health perceptions (five items); energy/vitality (four items); social functioning (two items); emotional role limitations (three items) and mental health (five items). Numeric values are recorded per scoring key and transformed into a lineal scale that was used to calculate the physical component summary (PCS) and mental component summary (MCS) [30]. As more generic QoL instruments might over- or underestimate the true impact of the disease and XLA patients might experience problems in similar health domains as patients with common variable immune deficiency (CVID), the 32-item CVID_QoL questionnaire was included as well [31]. The CVID_QoL questionnaire includes items in three dimensions: emotional functioning (EF) (13 items), relational functioning (RF) (9 items) and gastrointestinal/skin symptoms (GSS) (4 items). Each item is rated on a 5-point scale (0 =never, 4 =always). There are no negatively worded items, and higher values generally indicate higher degree of disability [32]. To include more disease specific questions, the PADQOL-16 instrument was additionally sent out to adult patients. This 16-item questionnaire was developed for adults with primary antibody immune deficiencies (PAD) and includes questions on patient’s self-perceived well-being that are related to PAD or treatment modalities. Health domains include general health, vitality, physical health, mental health, and social functioning. The survey is scored on a 3-point scale (0=never/rarely, 1=sometimes, 2=often/always) and items are summed for each scale to calculate a final total score for all items [33].

**Statistics**

Shapiro-Wilk test was used to determine whether data was normally distributed. Independent t-test was used to compare means in normally distributed data, whereas Mann-Whitney U was used as a non-parametric test to compare means ranks. Ordinal variables from scaled items in the questionnaire were reported as rating means. General population cohorts were chosen based on original publications of the standardized questionnaires. The Bonferroni test was used for multiple comparison correction.

The internal reality was determined by Cronbach's alpha for all questionnaires. A minimum standard of 0.70 for Cronbach's alpha coefficients was assumed for adequate internal consistency. Agreement between parent proxy-report child-self report was assessed with intraclass correlation coefficients.

Missing data were addressed according to the scoring algorithm of the standardized questionnaires. If more than 50% of the items in the PedsQL scale were missing, the scale score could not be computed. For the SF-36, items that are left blank are not taken into account when calculating the scale scores. Missing values were replaced with the respondent's mean score across the completed items in the same scale. For the CVID_QoL, scores of missing items were imputed as average scores for the same dimension when less than three answers were missing. In the parental questionnaire, missing data did not exceed 5%. P-values <0.05 were considered statistically significant.

**Results**

**Internal consistency of the QoL questionnaires**

Cronbach's alpha was used to test for internal consistency. All PedsQL self-report scales and parent-proxy scales exceeded the minimum reliability standard of 0.70 required for group comparison. Total PedsQL alpha coefficients were 0.956 and 0.922 respectively and exceeded the criterion of 0.90 recommended for analysis of individual patient scales. High internal consistency was found for the SF-36 questionnaire (Cronbach alpha 0.906). Cronbach alpha for the PADQOL-16 total score was 0.769 with an alpha coefficient in all subdomains >0.70. Internal consistency of the EF and RF subscales in the CVID_QoL was high (Cronbach alpha of 0.894 and 0.877 respectively). As previously observed, Cronbach alpha for the GSS subscale was 0.605, which would increase if item 26 “I felt uncomfortable because of my skin problems’ would be removed [31]. The Cronbach alpha for the parental questionnaire was 0.713 and the removal of three statements (B9, B12, B15 – see Questionnaire) would have resulted in a higher internal consistency and could therefore be considered for future studies.

**Tables**

**Table S1. QoL in health domains of the SF-36 reported by adult XLA patients**

| ***SF-36 health domains*** | **Total, N=88** | **Normative male population (N=1055) [33]** | **P-value** |
| --- | --- | --- | --- |
| Physical functioning, mean (SD) | 86.7 (19.8) | 87.2(21.3) | 0.838 |
| Physical limitations, mean (SD) | 77.2 (27.2) | 86.6 (30.9) | **<0.01** |
| Pain, mean (SD) | 76.3 (22.9) | 76.9 (23.0) | 0.820 |
| General Health, mean (SD) | 40.8 (24.1) | 73.5 (20.0) | **<0.01** |
| Energy Fatigue, mean (SD) | 53.2 (23.2) | 63.6 (20.0) | **<0.01** |
| Social functioning, mean (SD) | 76.8 (24.9) | 85.2 (21.3) | **<0.01** |
| Emotional limitations, mean (SD) | 76.3 (25.8) | 83.3 (31.3) | **0.04** |
| Emotional wellbeing, mean (SD) | 65.6 (21.0) | 76.4 (17.2) | **<0.01** |
| Psychical component summary, mean (SD) | 47.0 (10.6) | 50.0 (10.0) | **<0.01** |
| Mental component summary, mean (SD) | 45.2 (10.6) | 50.0 (10.0) | **<0.01** |

**Table S2. Pediatric self-report and parent-proxy QoL based on PedsQL Generic Core Scales.**

| **PedsQl domains** | **XLA patients N=65** | **General pediatric population [29]** | **P-value** |
| --- | --- | --- | --- |
| Self total score, mean (SD) | 77.4 (17.3) | 82.9 (13.2) | **<0.01** |
| Self total physical health, mean (SD) | 82.7 (17.9) | 86.9 (13.9) | **0.02** |
| Self psychosocial health, mean (SD) | 74.7 (18.4) | 80.7 (14.7) | **<0.01** |
| Self emotional functioning, mean (SD) | 71.2 (19.9) | 78.2 (17.4) | **<0.01** |
| Self social functioning, mean (SD) | 85.4 (21.4) | 84.0 (18.6) | 0.559 |
| Self school functioning, mean (SD) | 67.7 (21.6) | 79.9 (16.9) | **<0.01** |
| Parent total score, mean (SD) | 77.7 (14.4) | 81.3 (15.9) | 0.066 |
| Parent physical health, mean (SD) | 81.3 (18.2) | 83.3 (20.0) | 0.430 |
| Parent psychosocial health, core, mean (SD) | 75.2 (15.4) | 80.2 (15.8) | **0.01** |
| Parent emotional functioning ore, mean (SD) | 71.2 (18.1) | 80.3 (17.0) | **<0.01** |
| Parent social functioning, mean (SD) | 86.7 (17.4) | 82.2 (20.1) | 0.068 |
| Parent school functioning, mean (SD) | 68.3 (20.0) | 76.9 (20.2) | **<0.01** |

**Table S3. Personal quotes from parents on an early diagnosis of XLA and severe B-lymphocyte deficiencies**

|  | **Quotes from parents** |
| --- | --- |
| **1.** | *‘Early immunoglobulin replacement therapy can reduce the devastating effects of the disease’* |
| **2.** | *‘Not being exposed to unnecessary antibiotic drugs and radiation’* |
| **3.** | *Now damage to the lungs, eyes, and skin has occurred, which will not go away and we might have been able to prevent half of that, which would have been fantastic’* |
| **4.** | *‘Early diagnosis could prevent prolonged periods of illness and pain in these children, depriving them of part of their childhood. I still cry when I think about how sick our child was, fever for months on end. Before that, she had no energy to play and sat on the couch for hours’* |
| **5.** | *‘Early diagnosis would reduce the mental effects that occur for parents and children during the time leading up to the final diagnosis’* |
| **6.** | *‘Ignorance of the diagnosis before the impact of the disease affected the life of our entire family’* |
| **7.** | *‘I was heavily pregnant with our second son when our first son was diagnosed with XLA. It took another six months after his birth before it was confirmed from genetic research that our second son did not have XLA. It was a very uncertain period’* |
| **8.** | *‘Carrier girls could plan pregnancy and decide on their own whether or not to undergo timely therapy for their future sons’* |
| **9.** | *‘The earlier XLA is detected in a child, the earlier treatment is started and therefore the child's quality of life will not deteriorate’* |
| **10.** | *‘Limiting health damage, which makes the quality of life so much better for my child. Through NBS, the child does not build up fears due to frequent examinations, injections, etc. If diagnosed early, it is a "part of life" and the child will experience less mental burden as a result’* |
| **11.** | *‘As parents, you have more peace of mind if you know what is wrong with your child and you can also enjoy the time with your baby more instead of constantly being with a critically ill child and not knowing what is wrong with your child’.* |
| **12.** | *‘I hope that NBS for XLA will be available quickly so that others will be spared having to deal with a critically ill child for so long’* |
| **13.** | *‘Early diagnosis will increase awareness of these diseases among both doctors and society’* |
| **14.** | *‘XLA poses a diagnostic challenge due to the lack of specialist knowledge by most clinicians, the fact that children often are fine for the first year of life and symptoms present like common conditions such as cough/ear infections’* |
| **15.** | *‘As evident from my son’s case undiagnosed XLA can result in death. This can be avoided with NBS and early intervention. I believe that currently, we do not have true numbers of this condition as many children could be dying of “ sepsis” without a diagnosis of XLA due to delay. Without screening we are failing to help these children and save young lives’.* |
